# Clinical implications of changes in the diversity of *c-MYC* copy number variation after neoadjuvant chemotherapy in breast cancer

**DOI:** 10.1038/s41598-018-35072-5

**Published:** 2018-11-12

**Authors:** Yul Ri Chung, Hyun Jeong Kim, Milim Kim, Soomin Ahn, So Yeon Park

**Affiliations:** 1 Department of pathology, Seoul National University Bundang Hospital, Seongnam, Gyeonggi Republic of Korea; 2 Department of pathology, Seoul National University College of Medicine, Seoul, Republic of Korea

## Abstract

Chemotherapy can alter the makeup of a tumor cell population by exerting selection pressure. We examined the change in Shannon index, a mathematical diversity measure used in ecology, for *c-MYC* copy number variation (CNV) after neoadjuvant chemotherapy and evaluated its clinical significance in breast cancer. Associations between Shannon indices for *c-MYC* CNV in pre- and post-neoadjuvant chemotherapy breast cancer samples and clinicopathologic features of tumors as well as patient survival were analyzed in 144 patients. A change in *c-MYC* amplification and copy number gain status was found in 14.3% and 33.6% with most cases showing positive to negative conversion. The chemo-sensitive group showed a significant decrease in Shannon index after neoadjuvant chemotherapy. However, there was no difference in diversity indices between pre- and post-neoadjuvant chemotherapy specimens in the chemo-resistant group. In survival analyses, high Shannon indices for *c-MYC* CNV in post-neoadjuvant chemotherapy samples as well as those in pre-neoadjuvant chemotherapy samples were revealed as independent prognostic factors for poor disease-free survival not only in the whole group but also in the chemo-resistant subgroup. These findings suggest that a change in Shannon index for *c-MYC* CNV after neoadjuvant chemotherapy reflects chemo-responsiveness and that Shannon indices after neoadjuvant chemotherapy have a prognostic value in breast cancer patients who receive neoadjuvant chemotherapy.

## Introduction

Intratumoral heterogeneity refers to the presence of phenotypically and/or genetically distinct tumor cell populations within a tumor that may result in tumor progression^1,2^ and therapeutic resistance^3^. With recent technical advances including next generation sequencing, large amounts of data on intratumoral heterogeneity have accumulated, and our understanding of intratumoral heterogeneity and tumor evolution has increased. Most studies thus far have confirmed that intratumoral heterogeneity exists in many tumors, revealing driver genetic events and evolutionary mechanisms^4–8^. However, only a few have investigated the impact of intratumoral heterogeneity on treatment response or clinical outcome^9,10^, partly due to the difficulties with quantifying intratumoral heterogeneity via bioinformatics approach. Besides a whole genomic approach, a diversity index calculation-based approach using copy numbers of specific genetic loci is also promising in measuring intratumoral heterogeneity and correlating it with tumor progression^11–13^. Recently, we examined *c-MYC* copy number variation (CNV) in two cohorts of invasive breast cancer patients using an ecological diversity index, the Shannon index, and we found that a high Shannon index for *c-MYC* was a significant poor prognostic factor, which suggests that a diversity index of even a single gene can be a measure of intratumoral heterogeneity and can be used as a prognostic indicator^14^.

Intratumoral clonal heterogeneity can be altered when tumor cells undergo selection pressure driven by chemotherapy, the immune system, hypoxia, nutrient deprivation, or geographic isolation; the most potent selection pressure may be attributed to chemotherapy^15^. It has been suggested that chemotherapy imposes a bottleneck effect in the process of tumor progression^16^, which in turn, leads to a different composition of tumor cell populations in residual tumors after chemotherapy compared to those prior to treatment. However, comparative analyses of intratumoral heterogeneity in pre- and post-chemotherapy samples remain scarce in breast cancers. Previously, Almendro *et al*. analyzed cellular heterogeneity with genetic and phenotypic features as well as the spatial distribution of tumor cells in pre- and post-neoadjuvant chemotherapy breast cancer^17^. They reported that there was no change in genetic diversity after chemotherapy, contradicting the general concept that chemo-sensitive subclones regress and resistant ones persist after chemotherapy, thereby leading to decreased genetic diversity after treatment. Although it was a comprehensive study incorporating genetic and phenotypic diversity in breast cancer pre- and post-neoadjuvant chemotherapy, patient survival in relation to cellular diversity after chemotherapy was not evaluated.

Here, we focused on the relationship between chemo-responsiveness and the changes in diversity index during chemotherapy using *c-MYC* which is located at one of the most unstable chromosomal regions (8q24) and frequently harbors copy number gain or amplification in breast cancer regardless of subtype^18–20^, in an attempt to find the clinicopathologic significance of post-treatment genetic diversity.

## Results

### Clinicopathologic characteristics

Of the 144 patients, estrogen receptor (ER), progesterone receptor (PR), and human epidermal growth factor receptor 2 (HER2) were positive in 107 (74.3%), 90 (62.5%), and 31 (21.5%) patients, respectively. Luminal B subtype was the largest subtype comprising 44.4% of the cases. As for the chemotherapeutic regimen, 42 (29.2%) patients received the ‘AC’ regimen consisting of doxorubicin and cyclophophamide for 4 to 6 cycles, 70 (48.6%) patients received the ‘ACT’ regimen of 4 cycles of AC followed by 4 cycles of docetaxel, and lastly, the remaining 32 (22.2%) patients were treated with the ‘AD’ regimen of doxorubicin and docetaxel 3 to 6 cycles. Table 1 lists the baseline characteristics.

**Table 1:** Baseline characteristics of patients in pre-neoadjuvant chemotherapy status.

Characteristics	No. (%)
Age	
Mean ± SD	46.5 (±9.1)
Clinical stage	
II	65 (45.1)
III	79 (54.9)
Clinical T stage	
T1–T2	77 (53.5)
T3–T4	67 (46.5)
Clinical N stage	
N0	29 (20.1)
N1–N3	115 (79.9)
Histologic subtype	
IDC	129 (89.6)
ILC	6 (4.2)
Others	9 (6.2)
Histologic grade	
Low to intermediate	98 (68.1)
High	46 (31.9)
Estrogen receptor	
Positive	107 (74.3)
Negative	37 (25.7)
Progesterone receptor	
Positive	90 (62.5)
Negative	54 (37.5)
HER2 status	
Negative	113 (78.5)
Positive	31 (21.5)
Molecular subtype	
Luminal A	43 (29.9)
Luminal B	64 (44.4)
HER2+	15 (10.4)
Triple-negative	22 (15.3)
Ki-67 proliferation index	
<20%	65 (45.1)
≥20%	79 (54.9)
NAC regimen	
AC	42 (29.2)
AD	32 (22.2)
ACT	70 (48.6)

IDC, invasive ductal carcinoma; ILC, invasive lobular carcinoma; HER2, human epidermal growth factor receptor 2; NAC, neoadjuvant chemotherapy; AC, doxorubicin plus cyclophosphamide; AD, doxorubicin plus docetaxel; AC-T, AC followed by docetaxel.

### *c-MYC* CNV before and after neoadjuvant chemotherapy

In pre-neoadjuvant chemotherapy biopsy specimens, evaluation of *c-MYC* CNV was possible in 119 cases. *c-MYC* amplification was present in 18 (15.1%) cases while *c-MYC* copy number gain was present in 64 (53.8%) cases. Of the 144 post-neoadjuvant chemotherapy samples, *c-MYC* amplification and copy number gain were detected in 11 (7.6%) and 58 (40.3%) cases, showing decreased frequencies in post-neoadjuvant chemotherapy samples compared to pre-neoadjuvant chemotherapy samples (*P* = 0.054, *P* = 0.029, respectively). None of the cases showed *c-MYC* copy number loss in pre- and post-neoadjuvant chemotherapy samples.

**Figure 1:**
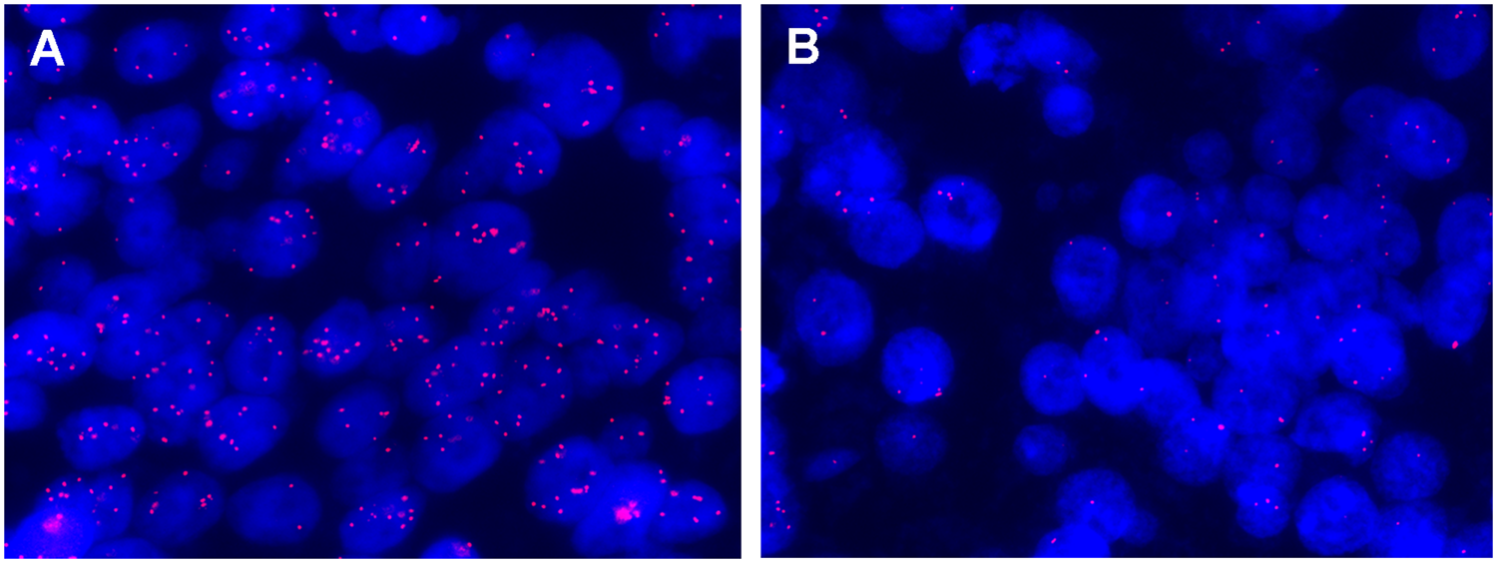
A representative case showing change in *c-MYC* amplification status after neoadjuvant chemotherapy. *c-MYC* amplification is present in the pre-neoadjuvant chemotherapy biopsy (**A**), but not in the post-neoadjuvant chemotherapy resection specimen (**B**).

Table 2 shows paired analyses of *c-MYC* CNV before and after neoadjuvant chemotherapy. Among the 119 patients in whom comparison between pre- and post-neoadjuvant chemotherapy samples was possible, a change in *c-MYC* amplification and copy number gain status between pre- and post-neoadjuvant chemotherapy specimens was found in 17 (14.3%) and 40 (33.6%) cases, respectively. Of the 17 patients with altered *c-MYC* amplification status, 13 showed conversion from amplified to non-amplified status, and 4 revealed conversion from non-amplified to amplified status (Fig. 1). All of the four cases showing alteration from a non-amplified to amplified status belonged to the chemo-resistant group (10.5% vs. 0%, chemo-resistant group vs. chemo-sensitive group; *P* = 0.009). Of the 40 patients with alterations in *c-MYC* copy number gain status, 30 showed changes from a positive to negative status, and 10 revealed changes from a negative to positive status.

**Table 2:** Paired analyses of *c-MYC* amplification and copy number gain before and after neoadjuvant chemotherapy.

	Group	Both (+)	Only pre-NAC (+)	Only post-NAC (+)	Both (−)
*c-MYC* amplification	Total (n = 119)	5 (4.2)	13 (10.9)	4 (3.4)	97 (81.5)
	Chemo-resistant (n = 38)	2 (5.3)	3 (7.9)	4 (10.5)^*^	29 (76.3)
	Chemo-sensitive (n = 81)	3 (3.7)	10 (12.3)	0 (0)^*^	68 (84.0)
*c-MYC* copy number gain	Total (n = 119)	34 (28.6)	30 (25.2)	10 (8.4)	45 (37.8)
	Chemo-resistant (n = 38)	13 (34.2)	6 (15.8)	3 (7.9)	16 (42.1)
	Chemo-sensitive (n = 81)	21 (25.9)	24 (29.6)	7 (8.6)	29 (35.8)

Numbers in parentheses indicate percentage.

NAC, neoadjuvant chemotherapy.

^*^P = 0.009.

### Shannon index for *c-MYC* CNV before and after neoadjuvant chemotherapy

**Figure 2:**
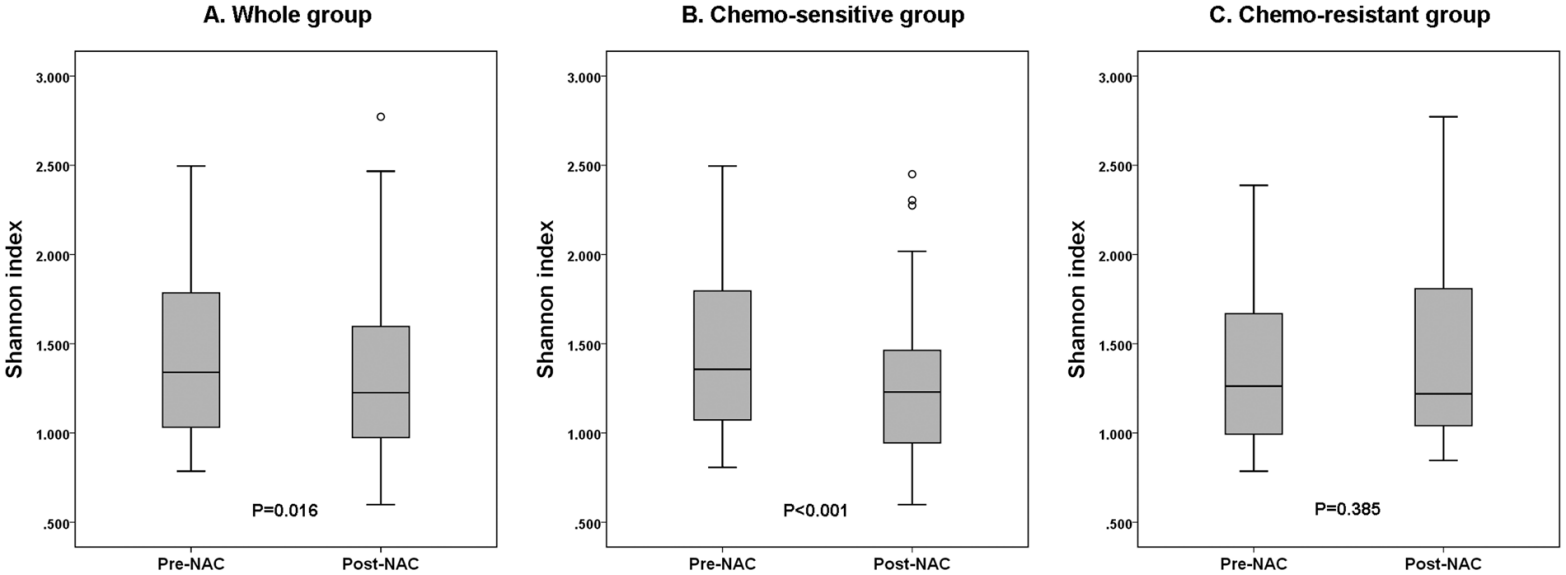
Change in Shannon indices for *c-MYC* copy number variation (CNV) after neoadjuvant chemotherapy according to chemo-responsiveness. (**A**) In the whole group, Shannon indices for *c-MYC* CNV after neoadjuvant chemotherapy (NAC) are significantly decreased compared to those before NAC. (**B**) In the chemo-sensitive group composed of Miller-Payne regression grade 3 and 4, Shannon indices are significantly decreased in post-NAC samples compared to pre-NAC samples. (**C**) In the chemo-resistant group including Miller-Payne regression grade 1 and 2, there is no difference between Shannon indices before and after NAC. The box shows the first to third quartiles and the horizontal line inside the box represents the median. The whiskers extend to minimum and maximum values within 1.5 times the interquartile range from the first and third quartiles, and the outliers are represented by small circles.

Shannon index calculated with *c-MYC* CNV ranged from 0.786 to 2.496 (median 1.340) in pre-neoadjuvant chemotherapy samples, and it ranged from 0.120 to 2.772 (median 1.231) in post-neoadjuvant chemotherapy samples. Shannon indices in pre- and post-ST samples showed a weak positive correlation (r = 0.431, *P* < 0.001; Supplementary Fig. S1). When we compared the changes in Shannon indices after neoadjuvant chemotherapy, they were decreased in the post-neoadjuvant chemotherapy samples compared to the pre-neoadjuvant chemotherapy samples as a whole (1.426 ± 0.443 vs. 1.321 ± 0.435, pre-neoadjuvant chemotherapy vs. post-neoadjuvant chemotherapy; *P* = 0.016, paired sample t-test; Fig. 2A). We also evaluated the change in Shannon indices according to chemo-responsiveness. The chemo-sensitive group showed a significant decrease in Shannon index after neoadjuvant chemotherapy (1.451 ± 0.441 vs. 1.266 ± 0.386, pre-neoadjuvant chemotherapy vs. post-neoadjuvant chemotherapy; *P* < 0.001, paired sample t-test; Fig. 2B) while the chemo-resistant group showed no difference in Shannon indices between pre- and post-neoadjuvant chemotherapy specimens (1.375 ± 0.449 vs. 1.440 ± 0.510; *P* = 0.385, paired sample t-test; Fig. 2C). However, there was no difference in Shannon indices after neoadjuvant chemotherapy between the chemo-resistant group and chemo-sensitive group (1.394 ± 0.573 vs. 1.287 ± 0.105; *P* = 0.242, independent sample t-test).

### Clinicopathological characteristics of tumors according to *c-MYC* CNV

We also evaluated the association between *c-MYC* CNV and clinicopathological characteristics of tumors before and after neoadjuvant chemotherapy (Supplementary Tables S1–S2). In pre-neoadjuvant chemotherapy status, *c-MYC* copy number gain was associated with high histologic grade (adj. *P* = 0.016), high Ki-67 index (adj. *P* = 0.004), and p53 overexpression (adj. *P* = 0.016). High Shannon index for *c-MYC* CNV (above median) in pre-neoadjuvant chemotherapy specimens was associated with high histologic grade (adj. *P* = 0.008), high Ki-67 index (adj. *P* = 0.016), and p53 overexpression (adj. *P* = 0.032). However, *c-MYC* amplification in pre-neoadjuvant chemotherapy samples was not associated with any of the clinicopathologic characteristics.

In post-neoadjuvant chemotherapy specimens, *c-MYC* amplification was associated with high yp T stage (adj. *P* = 0.027). *c-MYC* copy number gain in post-neoadjuvant chemotherapy specimens was associated with high histologic grade (adj. *P* < 0.001) and high Ki-67 index (adj. *P* = 0.009). High Shannon index (above median) in post-neoadjuvant chemotherapy specimens showed a significant association with high histologic grade (adj. *P* = 0.009) and high Ki-67 index (adj. *P* = 0.027).

### Prognostic significance of *c-MYC* CNV before and after neoadjuvant chemotherapy

**Figure 3:**
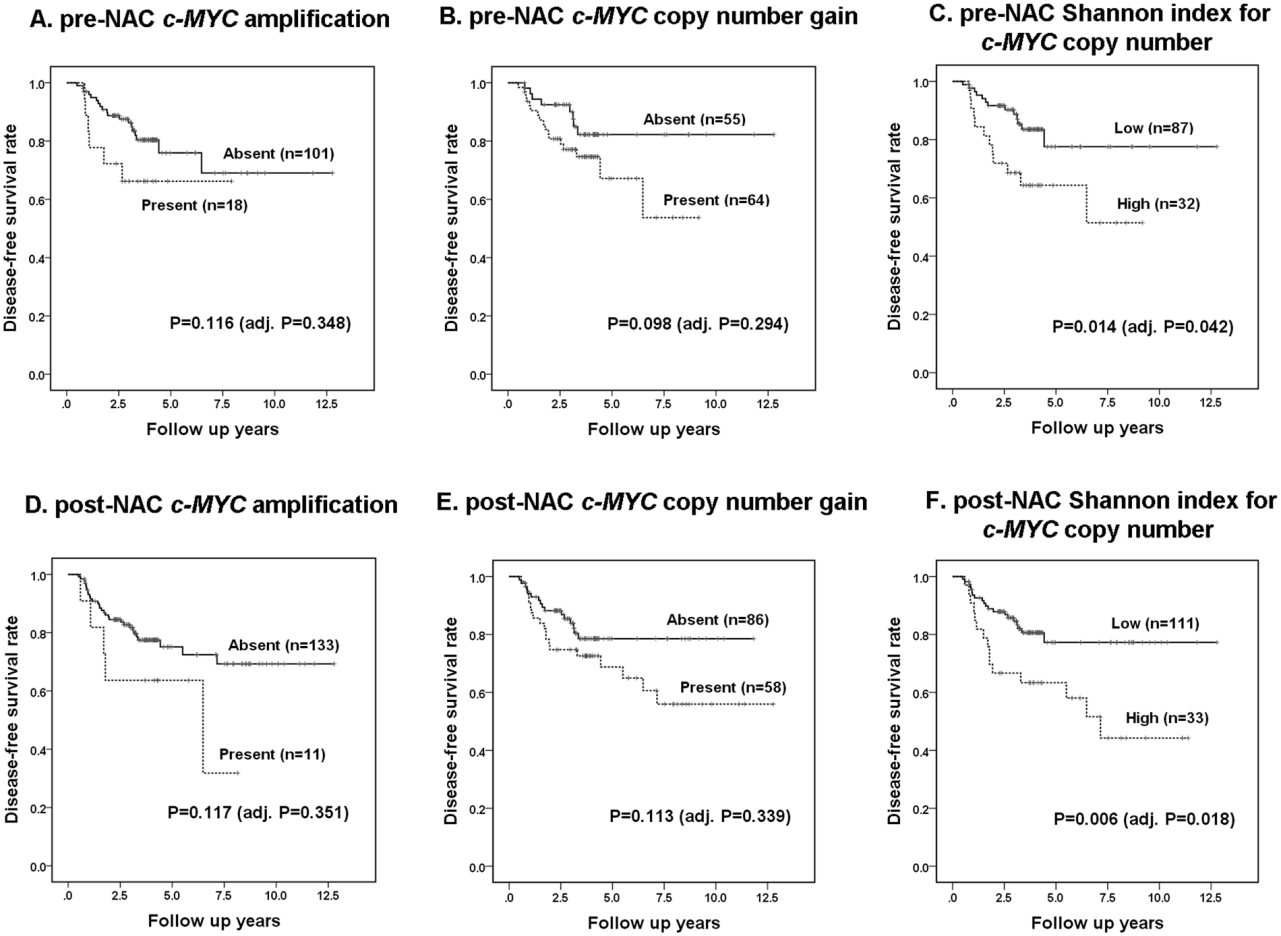
Kaplan-Meier survival analyses based on *c-MYC* copy number variation (CNV) before and after neoadjuvant chemotherapy. In the pre-neoadjuvant chemotherapy (NAC) samples, c*-MYC* amplification (**A**) and copy number gain (**B**) were not associated with disease-free survival of the patients, while a high Shannon index for *c-MYC* CNV (**C**) shows a significant association with poor disease-free survival. Similarly, in the post-NAC samples, *c-MYC* amplification (**D**) and copy number gain (**E**) were not associated with disease-free survival, and a high Shannon index (**F**) shows a significant association with poor disease-free survival.

Most of the patients were treated according to standard guidelines and were followed up regularly. One hundred and nineteen (82.6%) patients received radiation therapy, and 105 (72.9%) received endocrine therapy following surgery. The Kaplan-Meier plots for disease-free survival according to *c-MYC* CNV before and after neoadjuvant chemotherapy are shown in Fig. 3. In pre-neoadjuvant chemotherapy specimens, *c-MYC* amplification and copy number gain were not associated with disease-free survival of the patients (*P* = 0.116, *P = *0.098; adj. *P* = 0.348, adj. *P* = 0.294, respectively), and a high Shannon index using a cutoff value obtained by ROC analyses was significantly associated with poor disease-free survival (*P* = 0.014; adj. *P* = 0.042). In post-neoadjuvant chemotherapy specimens, a high Shannon index showed a significant association with poor disease-free survival (*P* = 0.006; adj. *P* = 0.018) and *c-MYC* amplification and copy number gain were not associated with disease-free survival of the patients (*P = *0.117, *P* = 0.113; adj. *P* = 0.351, adj. *P* = 0.339, respectively).

**Figure 4:**
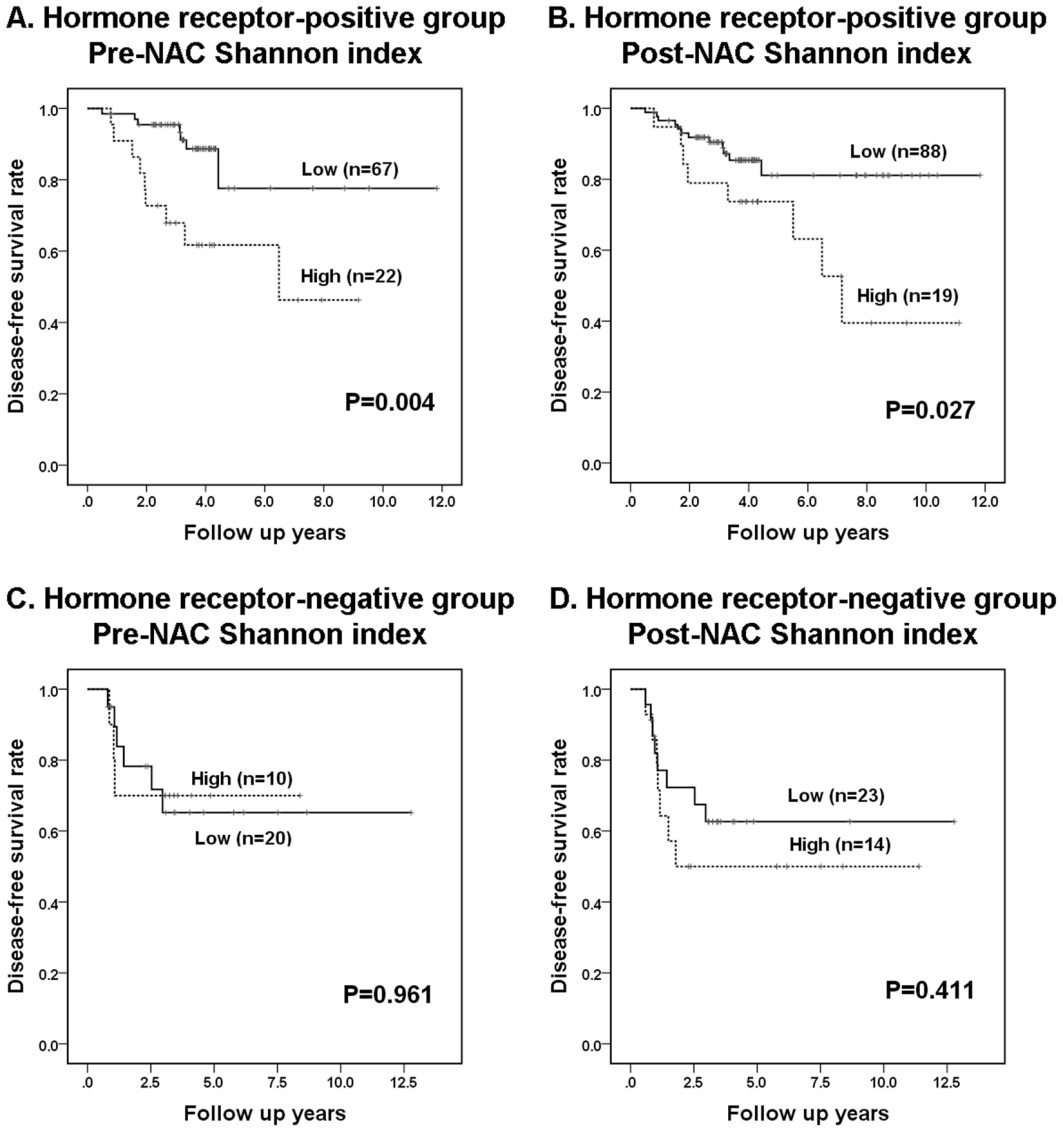
Kaplan-Meier survival analyses based on Shannon index for *c-MYC* copy number variation before and after neoadjuvant chemotherapy in subgroups according to hormone receptor status. In hormone receptor-positive subgroup, high Shannon indices before (**A**) and after (**B**) neoadjuvant chemotherapy (NAC) are associated with poor disease-free survival. On the contrary, in hormone receptor-negative subgroup, Shannon indices before (**C**) and after (**D**) NAC do not show association with patient survival.

Upon subgroup analysis according to hormone receptor status, high Shannon indices before and after neoadjuvant chemotherapy was associated with adverse clinical outcome only in hormone receptor-positive group (*P* = 0.004, *P* = 0.027, respectively; Fig. 4A,B) and not in hormone receptor-negative subgroup (*P* = 0.961, *P* = 0.411, respectively; Fig. 4C,D). In subgroup analysis by molecular subtype, high Shannon indices in pre-neoadjuvant chemotherapy samples were associated with poor disease-free survival (*P* = 0.012) in luminal B subtype, but those in post-neoadjuvant chemotherapy samples did not show an association with survival of the patients in any subtypes.

**Figure 5:**
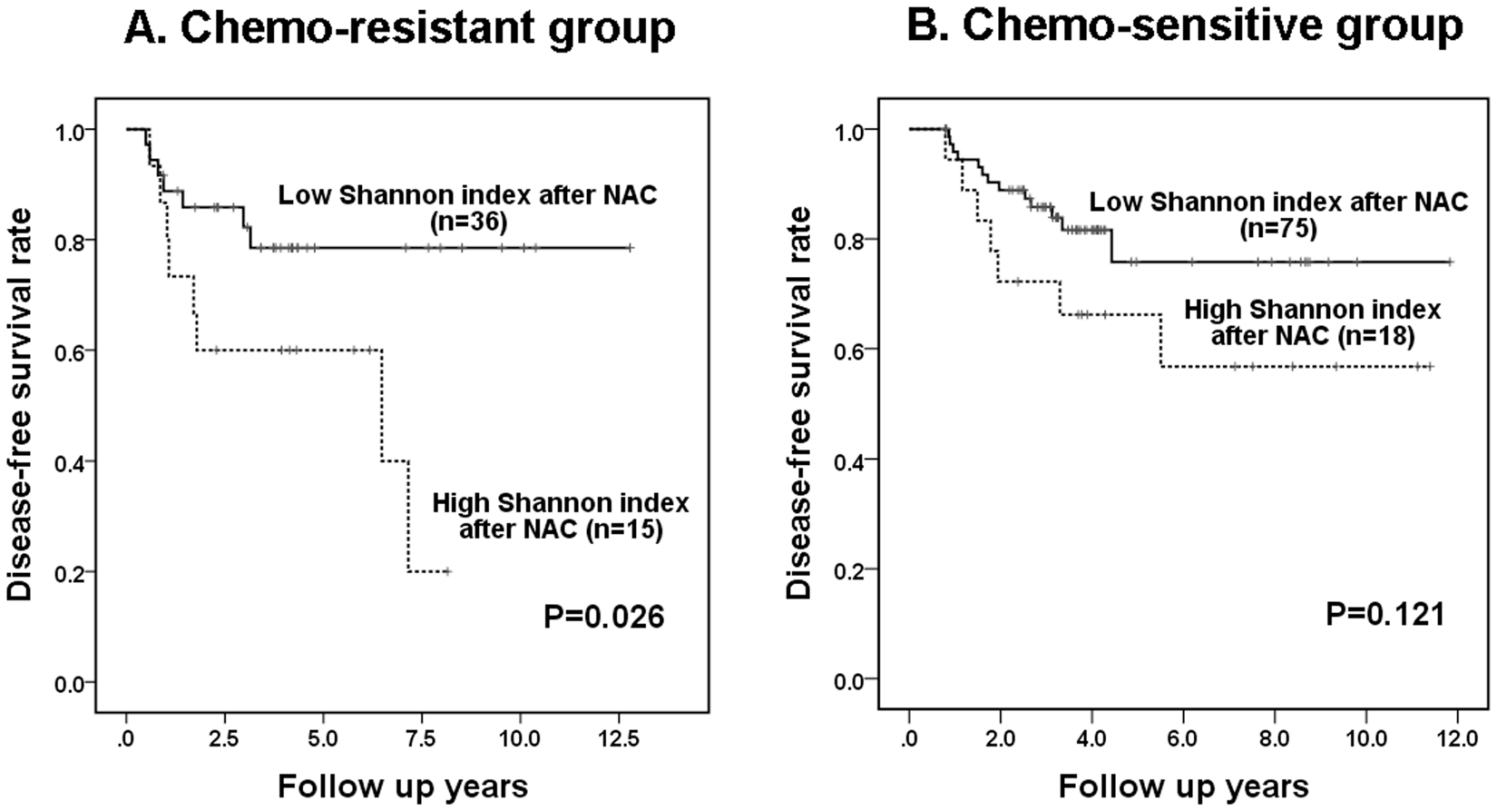
Kaplan-Meier survival analyses based on Shannon index for *c-MYC* copy number variation after neoadjuvant chemotherapy in subgroups according to chemo-responsiveness. (**A**) In chemo-resistant group, high Shannon index after neoadjuvant chemotherapy (NAC) shows a significant association with poor disease-free survival. (**B**) In chemo-sensitive group, it is not associated with disease-free survival of the patient.

In subgroup analyses according to chemo-responsiveness, a high Shannon index after neoadjuvant chemotherapy revealed an association with poor disease-free survival in the chemo-resistant group (*P* = 0.026; Fig. 5A), and it was not associated with survival in the chemo-sensitive group (*P* = 0.121; Fig. 5B).

Besides Shannon index for *c-MYC* CNV, high clinical N stage, pre- and post-neoadjuvant chemotherapy negative ER and PR status, and high Ki-67 index were poor prognostic indicators (Supplementary Table S3). In multivariate analyses, pre- and post-neoadjuvant chemotherapy high Shannon indices were revealed as adverse independent prognostic factors along with high N stage and negative hormone receptor status (Table 3).

**Table 3:** Multivariate analyses of disease-free survival.

	Variable	Category	Multivariate analysis		
			HR	95% CI	*P*-value
Pre-NAC status	cT stage	T1-2 vs. T3-4	1.057	0.458-2.441	0.896
	cN stage	N0 vs. N1-3	10.190	1.367–75.974	0.024
	ER status	Positive vs. Negative	1.556	0.508–4.761	0.439
	PR status	Positive vs. Negative	2.346	1.043–5.275	0.039
	Shannon index for *c-MYC* CNV	Low vs. High	2.342	1.059–5.182	0.036
	Ki-67 index	<20% vs. ≥20%	1.162	0.424–3.187	0.771
Post-NAC status	ypT stage	T1 vs. T2–4	1.515	0.687–3.344	0.303
	ypN stage	N0 vs. N1-3	2.638	1.067–6.519	0.036
	Miller-Payne grade	Grade 3–4 vs. 1–2	1.019	0.489–2.123	0.960
	ER status	Positive vs. Negative	3.400	1.695–6.822	0.001
	PR status	Positive vs. Negative	1.905	0.763–4.757	0.167
	Shannon index for *c-MYC* CNV	Low vs. High	2.122	1.071–4.204	0.031
	Ki-67 index	<20% vs. ≥20%	1.025	0.348–3.020	0.965

HR, hazard ratio; CI, confidence interval; NAC, neoadjuvant chemotherapy; ER, estrogen receptor; PR, progesterone receptor; CNV, copy number variation.

## Discussion

In locally-advanced breast cancer, neoadjuvant chemotherapy is considered standard treatment. The degree of response to neoadjuvant chemotherapy reflects chemo-sensitivity of a tumor to a certain chemotherapeutic agent and is associated with patient outcome; thus, patients who achieve pathologic complete response to neoadjuvant chemotherapy show excellent disease-free and overall survival^21,22^. With regards to intratumoral heterogeneity, a genetically homogenous population is expected to achieve complete response more frequently than genetically heterogeneous tumors. In residual disease after neoadjuvant chemotherapy, the partially-responsive tumors would remain as genetically homogeneous tumors due to a population bottleneck effect and reconstitution by the resistant clones while the resistant tumors would show no change in genetic diversity even after chemotherapy^23^. Thus, in this study, we tried to find the relationship between chemo-responsiveness and change in diversity index after neoadjuvant chemotherapy. To evaluate chemo-responsiveness, we adopted the Miller-Payne regression grading system^24^, which well matches the design of this study as it is based on the reduction in the proportion of tumor cellularity compared to pre-treatment primary tumor samples. We found that the Shannon index for *c-MYC* CNV generally decreased in the post-neoadjuvant chemotherapy samples compared to the pre-neoadjuvant chemotherapy samples in the whole group. In subgroup analyses according to chemo-responsiveness, the chemo-sensitive group showed a more significant decrease in Shannon index after neoadjuvant chemotherapy, whereas there was no difference in the Shannon indices between pre- and post-neoadjuvant chemotherapy samples in the chemo-resistant group. Thus, our study supports the current paradigm that a chemo-sensitive tumor is reconstituted by a relatively homogeneous tumor cell population and shows low genetic diversity after chemotherapy. More importantly, we showed that the change in Shannon index after neoadjuvant chemotherapy is indicative of chemo-responsiveness.

Previously, Almendro *et al*. analyzed diversity indices using probes for 8q24, 10p13, 16p13.3, and 20q13.31 in pre- and post-neoadjuvant chemotherapy samples of breast cancer patients; they showed that genetic diversity did not change after chemotherapy^17^. Chemotherapeutic agents cause DNA damage and cell cycle arrest, leading to genomic instability. Combined effects of decreased genetic diversity from selection pressure and increased genomic instability from genotoxicity of chemotherapeutic agents can result in no change in diversity index after chemotherapy. However, in their study, the number of cases was limited to only 43 cases, and the patients did not receive uniform treatment with some patients even receiving trastuzumab in combination with other chemotherapeutic agents. Moreover, they adopted clinical response over pathologic response in evaluation of chemoresponse. Generally, tumor cellularity decreases after chemotherapy; however, this decrease in tumor cellularity does not always result in a decrease in tumor size^25^. The clinical response to neoadjuvant chemotherapy evaluated by imaging studies such as MRI does not equate to the pathological response^26,27^. Only by pathologic examination of the residual tumors in surgically-resected specimens can a tumor’s chemo-sensitivity be accurately evaluated. For this reason, direct comparison of the results of the aforementioned study by Almendro *et al*. and ours is not feasible. A further large-scale study in an evenly-treated population is warranted to validate the results of this study.

In this study, we revealed the prognostic significance of high Shannon index for *c-MYC* CNV before and after neoadjuvant chemotherapy. The adverse prognostic value of high Shannon index prior to neoadjuvant chemotherapy is in line with our previous study^14^ which showed the prognostic value of high Shannon indices for gene CNV using *c-MYC* and *FGFR1* in treatment-naïve breast cancer patients. We believe this is the first study demonstrating the prognostic significance of post-neoadjuvant chemotherapy Shannon index using gene CNV in breast cancer. High Shannon index after neoadjuvant chemotherapy was proven an independent prognostic factor even after adjusting for Miller-Payne regression grade and post-neoadjuvant chemotherapy Ki-67 index, which are known to be associated with chemo-responsiveness and tumor recurrence after neoadjuvant chemotherapy^24,28,29^. Furthermore, the prognostic significance of high Shannon index after neoadjuvant chemotherapy was also found in the chemo-resistant subgroup.

In the post-neoadjuvant chemotherapy samples, high Shannon indices calculated with *c-MYC* CNV were associated with high Ki-67 index. As post-treatment Ki-67 is known to be higher in non-responders than responders^28^, the close relationship between Shannon index and Ki-67 index after neoadjuvant chemotherapy may suggest that a high Shannon index after neoadjuvant chemotherapy represents chemo-resistance. However, there was no statistical difference in Shannon indices after neoadjuvant chemotherapy between chemo-resistant and chemo-sensitive groups. Moreover, Shannon indices prior to and after neoadjuvant chemotherapy was commonly associated with adverse features of breast cancer including high histologic grade and high Ki-67 index, and they were correlated with each other. Thus, it seems that increased Shannon index after neoadjuvant chemotherapy not only represents chemo-resistance but also the intrinsic aggressiveness of the tumor, thereby encompassing poor prognostic impact in breast cancer patients who receive neoadjuvant chemotherapy.

In paired analyses of pre- and post-neoadjuvant chemotherapy breast cancer samples, a significant number of cases showed alterations in *c-MYC* amplification or copy number gain status (14.3% for amplification; 33.6% for copy number gain) with most cases showing positive to negative conversion. Therefore, it is probable that only the tumor cell populations with *c-MYC* amplification or copy number gain underwent complete response to chemotherapy, and the residual tumor showed no amplification or copy number gain. However, there were some cases that showed negative to positive conversion as well. Interestingly, all the cases that changed from a non-amplified to amplified status belonged to the chemo-resistant group, and *c-MYC* amplification after neoadjuvant chemotherapy was associated with high ypT stage. It is impossible for a tumor to have acquired *c-MYC* amplification or copy number gain during neoadjuvant chemotherapy if it responded to treatment. However, if the tumor were resistant to chemotherapy, it may be possible to acquire *c-MYC* amplification or copy number gain during neoadjuvant chemotherapy. Also, re-diversification may have occurred during the period from the end of neoadjuvant chemotherapy to surgery. Perhaps a more likely explanation may be that there existed primary intratumoral heterogeneity in *c-MYC* amplification or copy number gain in the treatment-naïve tumors, and sampling bias of the pre-neoadjuvant chemotherapy biopsy specimens caused the negative to positive conversion after neoadjuvant chemotherapy.

In conclusion, Shannon index for *c-MYC* CNV decreased after neoadjuvant chemotherapy especially in the chemo-sensitive group, and a post-treatment high Shannon index was found to be an independent poor prognostic indicator, suggesting that the change in Shannon index after neoadjuvant chemotherapy reflects chemo-responsiveness and that Shannon index after neoadjuvant chemotherapy can be used as a prognostic factor in patients with breast cancer who receive neoadjuvant chemotherapy.

## Methods

### Patients and tissue samples

This study was a retrospective one that included 144 patients with clinical stage II (*n* = 65) or III (*n* = 79) breast cancer. The patients received breast conserving surgery/mastectomy at Seoul National University Bundang Hospital between October 2004 and December 2012 following anthracycline/anthracycline and taxane-based neoadjuvant chemotherapy and had residual disease in the surgical resection specimen. Pre-chemotherapeutic core needle biopsy was performed prior to neoadjuvant chemotherapy, and all cases were diagnosed with invasive breast cancer. Surgery was performed 3–4 weeks after the last cycle of chemotherapy. For each patient, a pair of formalin-fixed and paraffin-embedded tumor samples from a pre-chemotherapy biopsy specimen and a post-chemotherapy resection specimen was retrieved. Pre-neoadjuvant chemotherapy biopsy specimen was not available in 25 patients whose biopsy was performed at an outside hospital or whose samples were too small for further study. As a result, comparison of pre- and post-neoadjuvant chemotherapy samples was performed in 119 patients. Clinicopathologic information including age, sex, initial clinical T stage and N stage, chemotherapeutic regimen, number of neoadjuvant chemotherapy cycles, pathologic T stage and N stage after neoadjuvant chemotherapy, histologic subtype, histologic grade, and lymphovascular invasion was obtained from the medical records and H&E-stained sections. Pathologic response to neoadjuvant chemotherapy was evaluated by Miller-Payne regression grading system^24^. Of the 144 post-neoadjuvant chemotherapy samples, 12 (8.3%) cases belonged to Miller-Payne regression grade 1, 39 (27.1%) to grade 2, 91 (63.2%) to grade 3, and the rest 2 (1.4%) to grade 4. Miller-Payne regression grades 1 and 2 were regarded as chemo-resistant, non-responder group, and Miller-Payne regression grades 3 and 4 were regarded as chemo-sensitive, responder group. The study was approved by the institutional review board of Seoul National University Bundang Hospital (protocol # B-1601/332-304), which waived the requirement for informed consent. All procedures performed in this study were in accordance with the ethical standards of the institutional research committee and with the 1964 Helsinki declaration and its later amendments or comparable ethical standards.

### Fluorescence *in situ* hybridization

Commercially available LSI *c-MYC* SpectrumOrange probe (8q24.12-q24.13) (Abbott Molecular, Downers Grove, IL, USA) was used for fluorescence *in situ* hybridization (FISH) of *c-MYC*. 4-μm-thick deparaffinized tumor tissue sections were incubated in pretreatment solution (Abbott Molecular) at 80 °C for 30 min first then in protease solution (Abbott Molecular) for 20 min at 37 °C. tDen-Hyb-2 hybridization buffer (InSitus Biotechnologies, Albuquerque, NM) was used for dilution of the probes. DNA denaturation was achieved by incubating the probes and the tissue sections in HYBriteTM (Abbott Molecular) for 5 min at 73 °C and hybridization at 37 °C for 16 hours. Post-hybridization washes were performed as per manufacturer’s instructions. Slides were mounted in 40, 6-diamidino-2-phenylindole/anti-fade, and a fluorescence microscope was used to view them.

The number of *c-MYC* signals per cell was counted in 100 tumor cells. *c-MYC* copy number gain was defined as an average copy number ≥3.0, *c-MYC* amplification as a copy number ≥6.0, and *c-MYC* copy number loss as a copy number < 1.6. To express diversity in *c-MYC* copy number per cell as a numerical value, we adopted the Shannon index, a diversity measure used in ecology, which estimates the number and distribution of species in a population. It is calculated as H’ = −∑ p_i_ ln(p_i_), where p_i_ equals the frequency of species i in the population^30^. A species, in this study, represents the tumor cells with the same copy number of *c-MYC and* p_i_ represents the proportion of tumor cells with the same copy number of *c-MYC*. The Shannon index appears as any non-negative value with a high value indicating high diversity.

### Immunohistochemical analyses of standard biomarkers

Expression of ER, PR, HER2, Ki-67, and p53 was evaluated at the time of diagnosis or during the study by immunohistochemistry (IHC). For ER and PR, positive expression was defined as nuclear staining in 1% or more tumor cells, and the expression level was recorded in 10% increments. HER2 considered positive when IHC revealed strong positive expression of 3+ or when gene amplification was observed in FISH for HER2 IHC 2+ cases. P53 overexpression was defined as positive staining in 10% or more tumor cells. Positive staining in 20% or more tumor cells was considered to have high Ki-67 proliferation index.

Classification of breast cancer subtypes was done in accordance with the 2011 St. Gallen Expert Consensus^31^: luminal A (ER+ and/or PR+, HER2−, Ki-67 < 14%), luminal B (ER+ and/or PR +, HER2−, Ki-67 ≥ 14%; ER+ and/or PR+, HER2+), HER2 + (ER−, PR−, HER2+), and triple-negative subtype (ER−, PR−, HER2−).

### Statistical analysis

Statistical package, SPSS version 21.0 for Windows (IBM Corp., ARMONK, NY) was used for statistical analysis. Pearson’s chi-square test was used to compare categorical variables between two groups. Continuous variables between two independent groups were compared with independent sample t-test. Paired sample t-test was used to compare continuous variables between pre- and post-neoadjuvant chemotherapy samples. Cut-off values for Shannon index that maximized the sum of sensitivity and specificity in outcome prediction were determined from receiver operating characteristic (ROC) analysis. Kaplan-Meier survival curves were drawn for survival analysis, and the difference was analyzed by log rank test. Multivariate analysis was performed using the Cox proportional hazards regression model with a backward stepwise selection method. Hazard ratios (HR) as well as 95% confidence intervals (CI) were calculated for the statistically significant variables. When needed, corrections for multiple testing were performed with Bonferroni method and adjusted (adj.) *P* values were presented. *P* values less than 0.05 were considered significant with all reported *P* values being two-sided.

## Electronic supplementary material

Supplementary Figure S1

Supplementary tables S1-S3

## Data Availability

The datasets used and/or analyzed during the current study are available from the corresponding author on reasonable request.
