# Supplementary material for: Clinical implications of changes in the diversity of c-MYC copy number variation after neoadjuvant chemotherapy in breast cancer
Source: Sci Rep. 2018 Nov 12;8:16668. doi: 10.1038/s41598-018-35072-5 (PMC6232091; doi:10.1038/s41598-018-35072-5)
Supplement: Supplementary file 1 — Supplementary Figure S1 [file 41598_2018_35072_MOESM1_ESM.pdf]

SUPPLEMENTARY INFORMATION

Clinical implications of changes in the diversity of *c-MYC* copy number variation after neoadjuvant chemotherapy in breast cancer

Yul Ri Chung[1, 2], Hyun Jeong Kim[1], Milim Kim[1, 2], Soomin Ahn[1], So Yeon Park[1, 2]

[1]Department of pathology, Seoul National University Bundang Hospital, Seongnam, Gyeonggi, Republic of Korea; [2]Department of pathology, Seoul National University College of Medicine, Seoul, Republic of Korea

Supplementary Figure S1. Correlation between Shannon indices for *c-MYC* copy number variation before and after neoadjuvant chemotherapy.

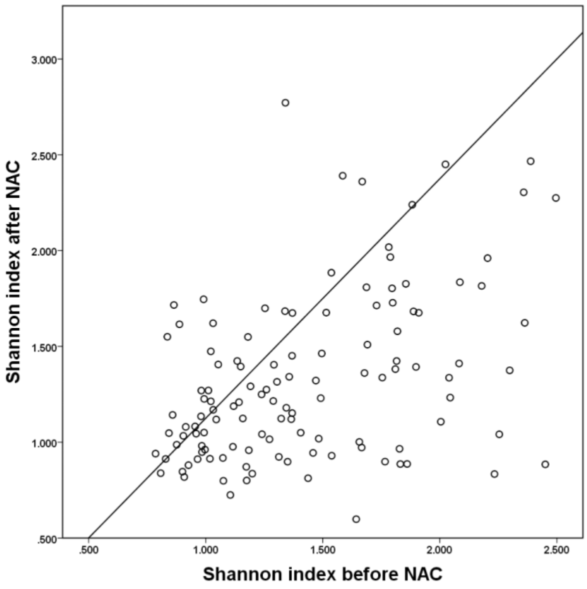

Shannon indices prior to neoadjuvant chemotherapy and those after neoadjuvant chemotherapy show a weak positive correlation (r=0.431, *P*<0.001).
