# Supplementary material for: Clinical implications of changes in the diversity of c-MYC copy number variation after neoadjuvant chemotherapy in breast cancer
Source: Sci Rep. 2018 Nov 12;8:16668. doi: 10.1038/s41598-018-35072-5 (PMC6232091; doi:10.1038/s41598-018-35072-5)
Supplement: Supplementary file 2 — Supplementary tables S1-S3 [file 41598_2018_35072_MOESM2_ESM.docx]

*SUPPLEMENTARY TABLES*

**Clinical implications of changes in the diversity of *c-MYC* copy number variation after neoadjuvant chemotherapy in breast cancer**

Yul Ri Chung^1, 2^, Hyun Jeong Kim^1^, Milim Kim^1, 2^, Soomin Ahn^1^, So Yeon Park^1, 2^

^1^Department of pathology, Seoul National University Bundang Hospital, Seongnam, Gyeonggi, Republic of Korea; ^2^Department of pathology, Seoul National University College of Medicine, Seoul, Republic of Korea

**Supplementary Table S1. Relationship between *c-MYC* copy number variation and clinicopathologic characteristics of tumors in pre-neoadjuvant chemotherapy specimens**

| Clinicopathologic  Characteristics before PST | *c-MYC* amplification | | adj. *P*-value | *c-MYC* copy number gain | | adj. *P*-value | Shannon index for *c-MYC* CNV | | adj. *P*-value |
| --- | --- | --- | --- | --- | --- | --- | --- | --- | --- |
|  | Absent | Present |  | Absent | Present |  | Low | High |  |
|  | No. (%) | No. (%) |  | No. (%) | No. (%) |  | No. (%) | No. (%) |  |
| cT stage |  |  | N. S. |  |  | N. S. |  |  | N. S. |
| T1-T2 | 59 (58.4) | 9 (50.0) |  | 34 (61.8) | 34 (53.1) |  | 39 (66.1) | 29 (48.3) |  |
| T3-T4 | 42 (41.6) | 9 (50.0) |  | 21 (38.2) | 30 (49.6) |  | 20 (33.9) | 31 (51.7) |  |
| cN stage |  |  | N. S. |  |  | N. S. |  |  | N. S. |
| N0 | 24 (23.8) | 3 (16.7) |  | 12 (21.8) | 15 (23.4) |  | 12 (20.3) | 15 (25.0) |  |
| N1-N3 | 77 (76.2) | 15 (83.3) |  | 43 (78.2) | 49 (76.6) |  | 47 (79.7) | 45 (75.0) |  |
| Histologic grade |  |  | N. S. |  |  | 0.016 |  |  | 0.008 |
| Low to intermediate | 70 (69.3) | 10 (55.6) |  | 45 (81.8) | 35 (54.7) |  | 48 (81.4) | 32 (53.3) |  |
| High | 31 (30.7) | 8 (44.4) |  | 10 (18.2) | 29 (45.3) |  | 11 (18.6) | 28 (46.7) |  |
| Estrogen receptor |  |  | N. S. |  |  | N. S. |  |  | N. S. |
| Negative | 25 (24.8) | 5 (27.8) |  | 10 (18.2) | 20 (31.3) |  | 10 (16.9) | 20 (33.3) |  |
| Positive | 76 (75.2) | 13 (72.2) |  | 45 (81.8) | 44 (68.8) |  | 49 (83.1) | 40 (66.7) |  |
| Progesterone receptor |  |  | N. S. |  |  | N. S. |  |  | N. S. |
| Negative | 38 (37.6) | 8 (44.4) |  | 17 (30.9) | 29 (45.3) |  | 17 (28.8) | 29 (48.3) |  |
| Positive | 63 (62.4) | 10 (55.6) |  | 38 (69.1) | 35 (54.7) |  | 42 (71.2) | 31 (51.7) |  |
| HER2 status |  |  | N. S. |  |  | N. S. |  |  | N. S. |
| Negative | 80 (79.2) | 12 (66.7) |  | 46 (83.6) | 46 (71.9) |  | 50 (84.7) | 42 (70.0) |  |
| Positive | 21 (20.8) | 6 (33.3) |  | 9 (16.4) | 18 (28.1) |  | 9 (15.3) | 18 (30.0) |  |
| Ki-67 index |  |  | N. S. |  |  | 0.004 |  |  | 0.016 |
| Low (<20%) | 44 (43.6) | 6 (33.3) |  | 33 (60.0) | 17 (26.6) |  | 33 (55.9) | 17 (28.3) |  |
| High (≥20%) | 57 (56.4) | 12 (66.7) |  | 22 (40.0) | 47 (73.4) |  | 26 (44.1) | 43 (71.7) |  |
| P53 overexpression |  |  | N. S. |  |  | 0.016 |  |  | 0.032 |
| Absent | 68 (67.3) | 12 (66.7) |  | 45 (81.8) | 35 (54.7) |  | 47 (79.7) | 33 (55.0) |  |
| Present | 33 (32.7) | 6 (33.3) |  | 10 (18.2) | 29 (45.3) |  | 12 (20.3) | 27 (45.0) |  |

CNV, copy number variation; N. S., not significant

Corrections for multiple testing are performed with Bonferroni method and adjusted (adj.) P values are presented.

**Supplementary Table S2. Relationship between *c-MYC* copy number variation and clinicopathologic characteristics of tumors in post-neoadjuvant chemotherapy specimens**

| Clinicopathologic  Characteristics after PST | *c-MYC* amplification | | adj. *P*-value | *c-MYC* copy number gain | | adj. *P*-value | Shannon index for *c-MYC* CNV | | adj. *P*-value |
| --- | --- | --- | --- | --- | --- | --- | --- | --- | --- |
|  | Absent | Present |  | Absent | Present |  | Low | High |  |
|  | No. (%) | No. (%) |  | No. (%) | No. (%) |  | No. (%) | No. (%) |  |
| ypT stage |  |  | 0.027 |  |  | N. S. |  |  | N. S. |
| T1 | 61 (45.9) | 0 (0.0) |  | 42 (48.8) | 19 (32.8) |  | 33 (45.8) | 28 (38.9) |  |
| T2-T4 | 72 (54.1) | 11 (100.0) |  | 44 (51.2) | 39 (67.2) |  | 39 (54.2) | 44 (61.1) |  |
| ypN stage |  |  | N. S. |  |  | N. S. |  |  | N. S. |
| N0 | 40 (30.1) | 2 (18.2) |  | 28 (32.6) | 14 (24.1) |  | 23 (31.9) | 19 (26.4) |  |
| N1-ypN3 | 93 (69.9) | 9 (81.8) |  | 58 (67.4) | 44 (75.9) |  | 49 (68.1) | 53 (73.6) |  |
| Histologic grade |  |  | N. S. |  |  | <0.001 |  |  | 0.009 |
| Low to intermediate | 90 (67.7) | 8 (72.7) |  | 70 (81.4) | 28 (48.3) |  | 58 (780.6) | 40 (55.6) |  |
| High | 43 (32.3) | 3 (27.3) |  | 16 (18.6) | 30 (51.7) |  | 14 (19.4) | 32 (44.4) |  |
| Miller-Payne grade |  |  | N. S. |  |  | N. S. |  |  | N. S. |
| Grade 1-2 | 43 (32.3) | 8 (72.7) |  | 29 (33.7) | 22 (37.9) |  | 27 (37.5) | 24 (33.3) |  |
| Grade 3-4 | 90 (67.7) | 3 (27.3) |  | 57 (66.3) | 36 (62.1) |  | 45 (62.5) | 48 (66.7) |  |
| Estrogen receptor |  |  | N. S. |  |  | N. S. |  |  | N. S. |
| Negative | 35 (26.3) | 3 (27.3) |  | 17 (19.8) | 21 (36.2) |  | 15 (20.8) | 23 (31.9) |  |
| Positive | 98 (73.7) | 8 (72.7) |  | 69 (80.2) | 37 (63.8) |  | 57 (79.2) | 49 (68.1) |  |
| Progesterone receptor |  |  | N. S. |  |  | N. S. |  |  | N. S. |
| Negative | 60 (45.1) | 5 (45.5) |  | 32 (37.2) | 33 (56.9) |  | 28 (38.9) | 37 (51.4) |  |
| Positive | 73 (54.9) | 6 (54.5) |  | 54 (62.8) | 25 (43.1) |  | 44 (61.1) | 35 (48.6) |  |
| HER2 status |  |  | N. S. |  |  | N. S. |  |  | N. S. |
| Negative | 102 (76.7) | 6 (54.5) |  | 67 (77.9) | 41 (70.7) |  | 55 (76.4) | 53 (73.6) |  |
| Positive | 31 (23.3) | 5 (45.5) |  | 19 (22.1) | 17 (29.3) |  | 17 (23.6) | 19 (26.4) |  |
| Ki-67 index |  |  | N. S. |  |  | 0.009 |  |  | 0.027 |
| Low (<20%) | 88 (66.2) | 7 (63.6) |  | 66 (76.7) | 29 (50.0) |  | 56 (77.8) | 39 (54.2) |  |
| High (≥20%) | 45 (33.8) | 4 (36.4) |  | 20 (23.3) | 29 (50.0) |  | 16 (22.2) | 33 (45.8) |  |
| P53 overexpression |  |  | N. S. |  |  | N. S. |  |  | N. S. |
| Absent | 91 (68.4) | 8 (72.7) |  | 62 (72.1) | 37 (63.8) |  | 54 (75.0) | 45 (62.5) |  |
| Present | 42 (31.6) | 3 (27.3) |  | 24 (27.9) | 21 (36.2) |  | 18 (25.0) | 27 (37.5) |  |

CNV, copy number variation; N. S., not significant

Corrections for multiple testing are performed with Bonferroni method and adjusted (adj.) P values are presented.

**Supplementary Table S3. Univariate analyses of disease-free survival**

|  | Variable | Category | Univariate analysis | | |
| --- | --- | --- | --- | --- | --- |
|  |  |  | HR | 95% CI | *P*-value |
| Pre-NAC status | cT stage | T1-2 vs. T3-4 | 1.506 | 0.766-2.960 | 0.235 |
|  | cN stage | N0 vs. N1-3 | 10.014 | 1.370-73.214 | 0.023 |
|  | ER status | Positive vs. Negative | 2.794 | 1.428-5.466 | 0.003 |
|  | PR status | Positive vs. Negative | 2.654 | 1.357-5.191 | 0.004 |
|  | Shannon index for *c-MYC* CNV | Low vs. High | 2.580 | 1.175-5.667 | 0.018 |
|  | Ki-67 index | <20% vs. ≥20% | 2.095 | 1.025-4.284 | 0.043 |
| Post-NAC status | ypT stage | T1 vs. T2-4 | 1.749 | 0.836-3.660 | 0.138 |
|  | ypN stage | N0 vs. N1-3 | 1.791 | 0.740-4.332 | 0.196 |
|  | Miller-Payne grade | Grade 3-4 vs. 1-2 | 1.423 | 0.728-2.783 | 0.302 |
|  | ER status | Positive vs. Negative | 3.114 | 1.599-6.067 | 0.001 |
|  | PR status | Positive vs. Negative | 2.747 | 1.366-5.523 | 0.005 |
|  | Shannon index for *c-MYC* CNV | Low vs. High | 2.482 | 1.266-4.865 | 0.008 |
|  | Ki-67 index | <20% vs. ≥20% | 2.284 | 1.176-4.436 | 0.015 |

HR, hazard ratio; CI, confidence interval; NAC, neoadjuvant chemotherapy; ER, estrogen receptor; PR, progesterone receptor; CNV, copy number variation
